# Coumarin Inhibits Primary Root Growth by Modulating Auxin Signaling via Neddylation

**DOI:** 10.3390/biology14121701

**Published:** 2025-11-28

**Authors:** Siqi Liu, Jie Li, Zixuan Zhao, Ting He, Hongxia Chang, Zhixuan Du, Longfei Zhu, Guanping Feng

**Affiliations:** 1 Key Laboratory of Jiangxi Province for Biological Invasion and Biosecurity, School of Life Sciences, Jinggangshan University, Ji’an 343009, China; 2 Key Laboratory of Jiangxi Province for Functional Biology and Pollution Control in Red Soil Regions, School of Life Sciences, Jinggangshan University, Ji’an 343009, China

**Keywords:** coumarin, neddylation, auxin signaling, primary root growth

## Abstract

**Simple Summary:**

Coumarin, a simple allelopathic compound found on various plant surfaces, facilitates inter-plant communication. Upon its release into the environment, it influences the growth and development of neighboring species. However, the underlying mechanism of this effect remains poorly understood. To investigate this molecular basis, we screened Arabidopsis mutants and isolated several mutants resistant to coumarin’s inhibition of root growth. Notably, these mutated genes are involved in ubiquitin-like modification and are key components of auxin signaling, implicating a direct link between coumarin’s action and auxin pathways. Elucidating this molecular mechanism is crucial for understanding plant competition and provides a theoretical foundation for agricultural applications.

**Abstract:**

The allelopathic compound coumarin inhibits root growth across numerous species, but its mechanism is unknown. Through a genetic screen in Arabidopsis mutants, we identified the mutants for *AXR1* (*AUXIN RESISTANT 1*) and *ECR1* (*E1 C-TERMINAL RELATED 1*), two subunits of the NEDD8-activating enzyme (NAE), that are resistant to coumarin. Conversely, overexpression of the NEDD8-encoding gene *RUB1 (RELATED TO UBIQUITIN 1)* caused hypersensitivity, while the NAE inhibitor MLN4924 blocked coumarin’s effect. Since neddylation regulates auxin signaling, we analyzed downstream AUX/IAA proteins and found that the loss-of-function mutant of *AUXIN RESISTANT 2* (*AXR2*) (also known as *IAA7*) was resistant to coumarin. We further showed that coumarin treatment leads to the accumulation of the AXR2 protein. Taken together, these results demonstrate that coumarin inhibits primary root growth by modulating auxin signaling via neddylation.

## 1. Introduction

Coumarin (1,2-benzopyrone), the simplest allelopathic compound among cinnamic acid derivatives, is widely distributed in many plant species and localized on the surfaces of leaves, seeds, flowers, and fruits, where it facilitates plant–plant communication [1]. When released into the environment, coumarin affects the growth and development of many plant species. Coumarin delays rice seed germination by suppressing abscisic acid catabolism and reactive oxygen species production [2,3]. Coumarins derived from plants modulate the composition of the Arabidopsis synthetic root microbial community (SynCom), potentially sculpting the root bacterial community by inhibiting the proliferation of a relatively abundant *Pseudomonas* species via a redox-mediated mechanism [4]. However, plant responses to coumarin vary by species, concentration, and physiological process, exhibiting stimulatory effects at low concentrations and inhibitory effects at high concentrations [2,5]. While coumarin’s inhibitory effects on cell division and root polarity in oat and *Phleum pratense* are well-documented, Neumann first reported its significant stimulatory effect on hypocotyl elongation in excised sunflower segments. Subsequent research has revealed that this stimulation occurs through the upregulation of brassinosteroid synthesis in plants [6,7].

Plants’ exploration and exploitation of soil resources (e.g., water, mineral nutrients) depend on root development [8]. Coumarin enhances nitrate uptake in maize roots by modulating plasma membrane H^+^-ATPase activity [9]. Recent research has identified coumarin exudation as a key mechanism facilitating plant iron acquisition [10,11]. The root system is an extremely plastic organ, adapting to external cues such as nutrient availability, soil moisture, matrix conditions, and allelopathic compounds [12,13]. As an active allelochemical, coumarin disrupts root morphology and histology, indicating the root system is its primary target, compromising both form and function [14]. Additionally, studies revealed that at low concentrations, coumarin specifically promoted lateral root elongation in Arabidopsis [15]. Combined morphological and electrophysiological approaches revealed that the root apex of maize primary roots exhibits the highest sensitivity to coumarin, suggesting auxin involvement in this response [16]. Genetic evidence from Arabidopsis auxin signaling mutants reveals that coumarin remodels root system architecture by functionally modulating polar auxin transport [17]. Although substantial evidence indicates coumarin’s effects on plant growth and development are closely linked to auxin function, the precise nature of this association remains poorly understood and requires further investigation.

Therefore, leveraging coumarin’s inhibitory effect on primary root growth in Arabidopsis, we conducted large-scale mutant screening and identified multiple auxin signaling-related mutants exhibiting resistance to coumarin. This finding not only further validated the relationship between coumarin and auxin but also enabled us to elucidate the molecular mechanism underlying coumarin’s allelopathic effects through auxin signaling pathways, employing molecular and cell biology techniques.

## 2. Materials and Methods

### 2.1. Plant Materials and Growth Conditions

This study utilized *Arabidopsis thaliana* ecotype Columbia-0 (Col-0) seeds from our laboratory collection, along with the mutant lines *axr1-12* (N3076), *axr1-3* (N3075), *axr2-1* (N3077) and *ecr1-1* (SALK_119046C) obtained from the Nottingham Arabidopsis Stock Centre (NASC) at the University of Nottingham, UK. Seeds were surface-sterilized and sown on half-strength Murashige and Skoog (1/2MS) medium supplemented with 1% (*w*/*v*) sucrose and solidified with 0.6% (*w*/*v*) agar. The plates were then incubated in a growth chamber under a 16-h light/8-h dark photoperiod at 22 ± 1 °C, with a light intensity of 80–90 µmol m^−2^s^−1^ to facilitate germination and seedling growth.

### 2.2. Cytological Observation and Tissue Staining

For GUS staining, samples were incubated in GUS staining solution containing 1 mM X-Gluc, 0.1% Triton X-100, 2 mM potassium ferricyanide, and 2 mM potassium ferrocyanide in 50 mM sodium phosphate buffer (pH 7.0). Vacuum infiltration was applied for 15 min, followed by incubation at 37 °C overnight in the dark. The staining solution was then replaced with 70% ethanol to clear chlorophyll. For DAB (3,3′-Diaminobiphenylamine) staining, duckweed and Arabidopsis seedlings were first incubated in a solution containing 0.1% DAB and 50 mM Tris-HCl (pH 5.0) for up to 30 min, adjusting the time for optimal staining. The seedlings were then cleared in 95% ethanol for 1 h before being mounted on glass slides with HCG solution (24 g chloral hydrate, 3 mL glycerol, 9 mL H_2_O). Imaging was performed using a Leica DM2500 microscope, Wetzlar, Germany.

### 2.3. Gene Expression Analysis

For the quantification of gene expression, total RNA was initially purified from 10-day-old Arabidopsis seedlings or roots via the TaKaRa MiniBEST Plant RNA Extraction Kit, Osaka, Osaka Prefecture, Japan. This RNA served as the template for first-strand cDNA synthesis, which was accomplished using the PrimeScript™ 1st Strand cDNA Synthesis Kit. Subsequently, the cDNA samples were analyzed by quantitative real-time PCR (qPCR) on a Thermo Fisher QuantStudio 3 system with the SYBR Premix Ex Taq™ II kit, Osaka, Osaka Prefecture, Japan. The ΔCt comparative method was employed to determine relative transcript levels, which were normalized against the internal reference gene *ACTIN2*. The sequences for all primers utilized in this study are provided in Table S1.

### 2.4. Plant Transformation

The full-length coding sequence (CDS, 471 bp) of *RUB1* was amplified via PCR and subsequently integrated into the binary vector pCambia1300, driven by the CaMV *35S* promoter, to generate the *35S-RUB1* overexpression construct for Arabidopsis transformation. For the construction of AXR2-Flag transgenic plants, the stop-codon-less CDS of *AXR2* (729 bp) was PCR-amplified and cloned into the pCambia1306-Flag vector. T1 generation seeds were subsequently screened on 1/2MS medium supplemented with hygromycin, and more than 20 positive seedlings were selected for phenotypic analysis.

### 2.5. Immunoblot Analysis

Total proteins were extracted from 10-day-old seedlings in RIPA buffer with protease inhibitors, and concentrations were measured by the BCA method. For immunoblotting, 20–40 µg of protein was resolved by 10% SDS-PAGE and transferred to a PVDF membrane. The membrane was blocked with 5% non-fat milk and probed with an anti-FLAG M2 monoclonal antibody (1:1000; Sigma-Aldrich, St. Louis, MO, USA), followed by an HRP-conjugated secondary antibody (1:5000; Cell Signaling Technology, Danvers, MA, USA. Signals were detected using an ECL system. Anti-ACTIN was used as a loading control.

## 3. Results

### 3.1. Coumarin Inhibits Primary Root Growth

**Figure 1:**
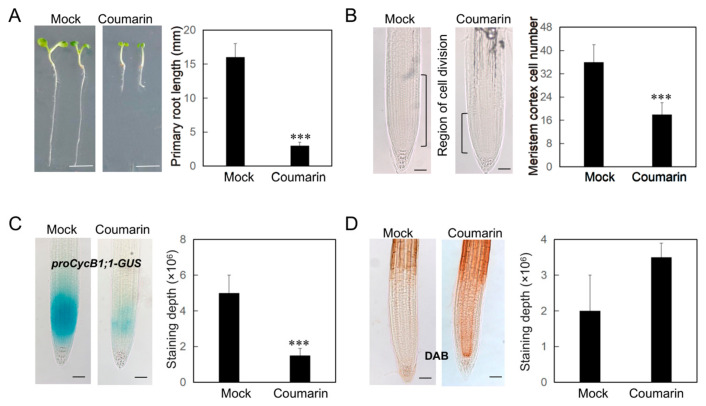
Coumarin suppresses primary root growth. (**A**) Phenotypes of wild-type (Col-0) Arabidopsis plants grown on 1/2MS medium with or without (Mock) 20 µM coumarin. Scale bar = 5 mm. Data are presented as the mean ± SE (n > 20). Asterisks indicate statistically significant differences (***, *p* < 0.01). (**B**) The primary root microstructures and the root meristem length after 20 µM coumarin treatment. Data are presented as the mean ± SE (n > 10). Asterisks indicate statistically significant differences (***, *p* < 0.01). (**C**) GUS staining of *proCycB1;1-GUS* plants treated after 1 days of 20 µM coumarin treatment. Scale bar = 100 µm. Data are presented as the mean ± SE (n > 10). Asterisks indicate statistically significant differences (***, *p* < 0.01). (**D**) DAB staining of Col-0 plantsafter 2 h of 20 µM coumarin treatment. Scale bar = 100 µm. Data are presented as the mean ± SE (n > 10).

As a classic allelochemical, coumarin is known to suppress seed germination and plant growth. To elucidate the molecular mechanism underlying this growth inhibition, we first analyzed the effects of coumarin at a range of concentrations. Notably, a concentration of just 20 µM was sufficient to cause a significant inhibition of primary root elongation in the model plant *Arabidopsis thaliana* (Figure 1A, Figure S1). Cytological analysis revealed that coumarin markedly suppressed cell division in the root tip, resulting in a root apical meristem that was half the length of the control (Figure 1B). Using the *proCycB1*;*1-GUS* cell division marker line, we found that coumarin treatment severely inhibited cell division in the root tip (Figure 1C). Additionally, DAB staining indicated an abnormal accumulation of hydrogen peroxide (H_2_O_2_) in the root apical meristem following coumarin treatment, with this effect being especially pronounced in the stele cells (Figure 1D). Collectively, these findings indicate that coumarin’s inhibition of root growth is mediated through the disruption of cell division.

### 3.2. The AXR1 Mutation Confers Resistance to Coumarin

**Figure 2:**
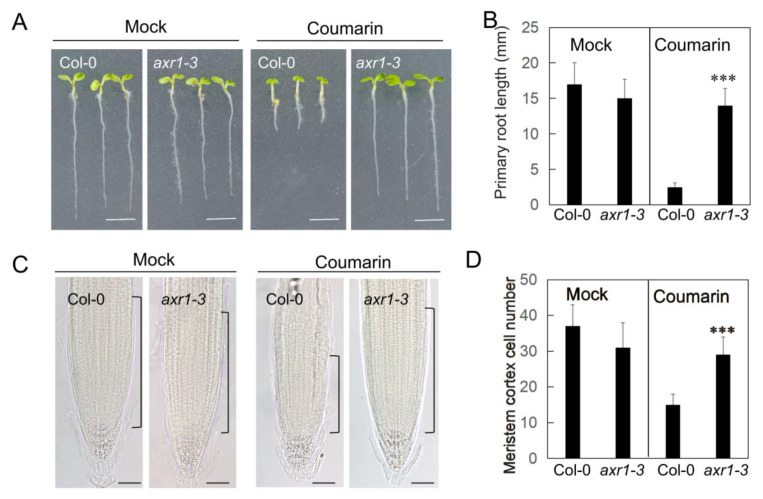
The *axr1-3* mutant is insensitive to coumarin. (**A**) Phenotypes of Col-0 and *axr1-3* plants grown on 1/2MS medium with or without (Mock) 20 µM coumarin. Scale bar = 5 mm. (**B**) The primary root length of plants in (**A**). Data are presented as the mean ± SE (n > 20). Asterisks indicate statistically significant differences (***, *p* < 0.01). (**C**) The primary root microstructures of plants in (**A**) after 20 µM coumarin treatment. (**D**) The meristem cortex cell number of plants in (**C**). Data are presented as the mean ± SE (n > 10). Asterisks indicate statistically significant differences (***, *p* < 0.01).

After establishing a model for the inhibitory effect of coumarin on *Arabidopsis thaliana* root growth, we screened an Arabidopsis mutant involved in phytohormone biosynthesis and signaling to identify mutants with significantly altered sensitivity to coumarin. Among the several candidate mutants identified, the *axr1-3* mutant exhibited nearly complete insensitivity (Figure 2A). On 1/2 MS medium containing 20 µM coumarin, the root length of wild-type (Col-0) seedlings was only 18% of that of the mock control, whereas the *axr1-3* mutant’s root length reached 95% of its control (Figure 2). The core function of the *AXR1* (*AUXIN RESISTANT 1*) is to regulate the auxin signaling pathway, and its loss-of-function mutants exhibit significant auxin resistance. These results indicate that the loss of *AXR1* function almost completely blocks the inhibitory effect of coumarin on root growth.

### 3.3. Coumarin Inhibition of Root Growth Requires NEDD8-Neddylation

**Figure 3:**
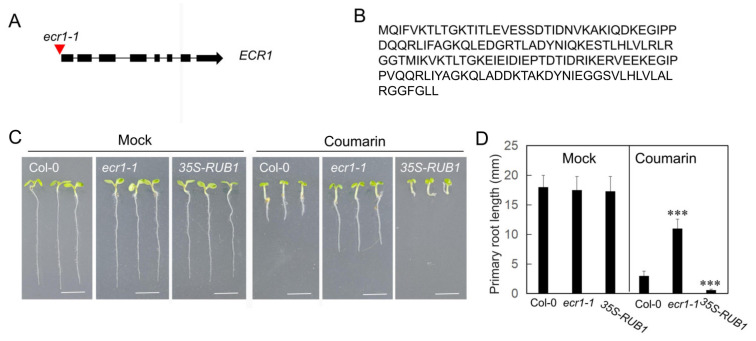
The inhibition of primary root growth by coumarin depends on neddylation. (**A**) Schematic diagram of *ECR1* and *ecr1-1* mutant. Red triangle marks the T-DNA insertion site (**B**) Amino acid sequence of RUB1 protein. (**C**) Phenotypes of Col-0, *ecr1-1* and *35S-RUB1* plants grown on 1/2MS medium with or without (Mock) 20 µM coumarin. Scale bar = 5 mm. (**D**) The primary root length of plants in (**C**). Data are presented as the mean ± SE (n > 20). Asterisks indicate statistically significant differences (***, *p* < 0.01).

Neddylation, the reversible conjugation of the ubiquitin-like protein NEDD8, is a well-characterized post-translational modification that primarily targets Cullin-RING E3 ligases (CRLs). Formation of the NEDD8 activating enzyme (NAE) requires heterodimerization between AXR1 and either E1 C-TERMINAL RELATED 1 (ECR1). Given that the *axr1-3* mutant is completely insensitive to coumarin, we hypothesized that the inhibition of root growth by coumarin depends on NEDD8-neddylation. To test this hypothesis, we analyzed the *ecr1-1* loss-of-function mutant from the NASC collection and found it was insensitive to coumarin, similar to the *axr1-3* mutant (Figure 3, Figure S2). Conversely, we generated transgenic lines overexpressing *RELATED TO UBIQUITIN 1* (*RUB1*) by placing its coding sequence under the control of the *35S* promoter. Analysis of the coumarin response revealed that these transgenic lines were hypersensitive to coumarin (Figure 3, Figure S2). These results show that NEDD8-neddylation is required for the coumarin-induced inhibition of primary root growth.

### 3.4. Convergent Inhibition of Root Growth by Coumarin and MLN4924

**Figure 4:**
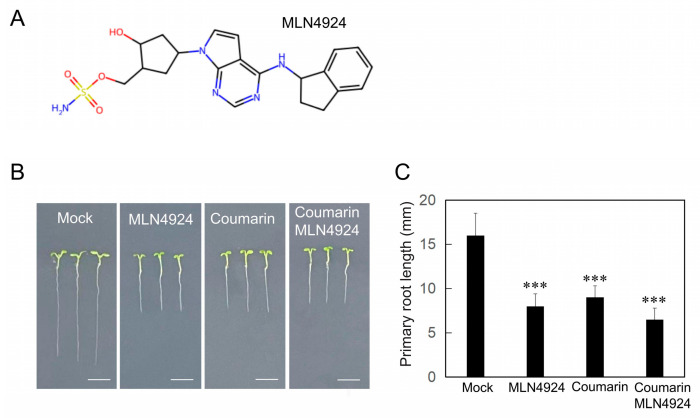
Inhibition of root growth by coumarin and MLN4924 via a common pathway. (**A**) MLN4924 chemical structure. (**B**) Phenotypes of Col-0 plants grown on 1/2MS medium with or without (Mock) 2 µM MLN4924 and/or 10 µM coumarin. (**C**) The primary root length of plants in (**B**). Data are presented as the mean ± SE (n > 20). Asterisks indicate statistically significant differences (***, *p* < 0.01).

Neddylation, the reversible conjugation of the ubiquitin-like MLN4924, a characterized inhibitor of human NAE, is a promising candidate drug for treating mammalian cancers. Structural studies have elucidated its mechanism of action, revealing it to be a mechanism-based inhibitor that forms a NEDD8-AMP mimic (Figure 4A). Hakenjos et al. reported that MLN4924 is also an effective and specific inhibitor of NAE1 enzymes in Arabidopsis and other plant species [18,19]. Both 2 µM MLN4924 and 10 µM coumarin strongly inhibited Arabidopsis root growth, reducing root length to 51% and 56% of the control, respectively. However, their combined treatment did not result in a further decrease in root length, showing an inhibitory effect significantly less than additive (Figure 4B,C). This indicates that coumarin and MLN4924 likely act within the same pathway to suppress root growth.

### 3.5. Coumarin Modulates the Degradation of AUX/IAA Protein

**Figure 5:**
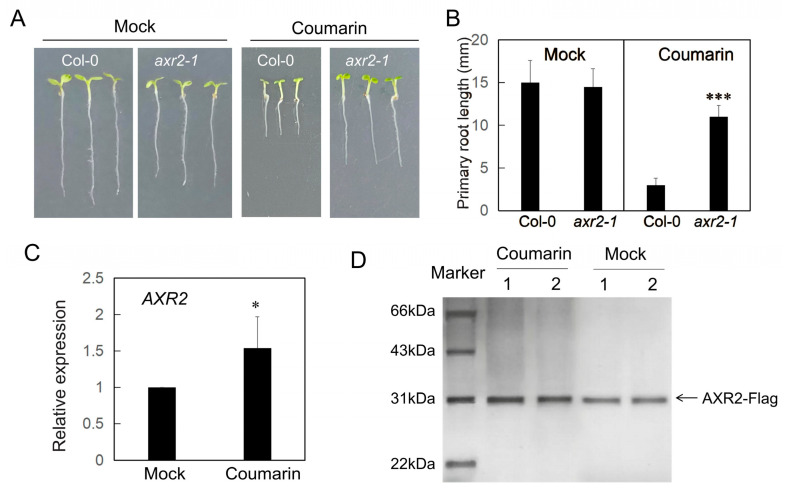
Degradation of the AXR2 is affected by coumarin. (**A**) Phenotypes of Col-0 and *axr2-1* plants grown on 1/2MS medium with or without (Mock) 20 µM coumarin. (**B**) The primary root length of plants in (**A**). Data are presented as the mean ± SE (n > 20). Asterisks indicate statistically significant differences (***, *p* < 0.01). (**C**) qPCR analysis of *AXR2* expression in the plants after 2 h of 20 µM coumarin treatment. The data show one of three independent experiments and error bars represent the standard error. Asterisks indicate statistically significant differences (*, *p* < 0.05) (**D**) AXR2 protein abundance was analyzed by Western blot in plants treated with 20 µM coumarin for 1 day (n = 2) and in mock-treated controls (n = 2).

Neddylation functions as a pivotal upstream regulatory event by modifying and activating the SCF ubiquitin ligase complex, a prerequisite for the selective degradation of AUX/IAA proteins. This process is essential for the swift and precise transduction of the auxin signal. Given that the *axr1-3* mutant is insensitive to both auxin and coumarin, and that AXR1 is a core component of the NEDD8-activating enzyme (NAE), we hypothesized that the inhibition of root growth by coumarin depends on the degradation of AUX/IAA proteins. To test this, we obtained mutants of AUX/IAAs and found that the loss-of-function mutant of (*AUXIN RESISTANT 2*) *AXR2* (also known as *IAA7*) was insensitive to coumarin (Figure 5A,B). Quantitative real-time PCR (qRT-PCR) analysis showed that coumarin treatment slightly induced *AXR2* expression (Figure 5C). Furthermore, we generated an AXR2-Flag fusion construct to monitor its protein abundance (Figure 5D). The results indicated that coumarin treatment led to an accumulation of AXR2 protein in planta. These results suggest that the inhibitory effect of coumarin on root growth relies on the degradation of AUX/IAA proteins.

## 4. Discussion

Coumarin is a potent allelopathic compound that plays a key role in plant–plant interactions by influencing the root growth of many species, although its mechanism of action is not well understood [17]. In this study, we found that treating *Arabidopsis thaliana* with 20 µM coumarin was sufficient to significantly inhibit primary root growth. Cytological observations further revealed that coumarin suppresses cell division in the root tip, which dramatically shortens the meristem zone and consequently affects primary root elongation. These results are consistent with findings from Goodwin and Avers, who reported similar effects in oat and Phleum pratense, indicating that the root growth-inhibiting action of coumarin is likely a conserved mechanism [20,21]. Previously, Pergo et al. [22] observed that coumarin induces abnormal ROS accumulation in the roots of *Bidens pilosa*. However, our cytological analysis refined this observation, showing that the accumulation was primarily due to a buildup of hydrogen peroxide (H_2_O_2_) within the stele cells. This finding offers a more precise understanding of the cellular mechanism underlying coumarin’s allelopathic action.

Neddylation, a critical post-translational modification that activates CULLIN-RING-type E3 ubiquitin ligases (CRLs), was first identified in Arabidopsis *axr1* mutants with impaired auxin responses [23,24,25]. The Arabidopsis NEDD8-activating enzyme (NAE) is a heterodimer composed of AXR1 and ECR1, which activates the NEDD8 modifier [26,27,28]. Consistent with this essential role, *axr1* and *ecr1* mutants are insensitive to auxin and display diminished root gravitropism [23,29]. We now show that these mutants are also completely insensitive to coumarin-mediated root growth inhibition, indicating that this effect requires NAE activity. Consistent with this, simultaneous treatment with the NAE inhibitor MLN4924 and coumarin failed to produce an additive inhibitory effect, strengthening the hypothesis that coumarin acts on NAE. While the root-inhibiting properties of coumarin have been long known, our study is the first to demonstrate that its allelopathic effect is mediated through the NEDD8-neddylation pathway.

Our study reveals a direct mechanistic link between coumarin and the auxin signaling pathway. We demonstrate that *axr1-3* and *axr2-1* mutants, which are insensitive to auxin, are also resistant to coumarin. This indicates that coumarin’s inhibitory effect operates through the core auxin pathway, which relies on neddylation to activate the SCF complex and trigger the selective degradation of AUX/IAA proteins. The *axr1-3* mutant is completely insensitive to the coumarin-induced inhibition of root growth, while the *axr2-1* mutant is only partially insensitive. This suggests that the function of coumarin in inhibiting root growth depends on neddylation, which affects the degradation of AUX/IAA proteins. Since AXR2 is just one of many AUX/IAA proteins targeted for neddylation-dependent degradation, it is possible that coumarin also affects the degradation of other AUX/IAA proteins. Furthermore, we show this process depends on the core NAE enzyme and that coumarin directly interferes with AUX/IAA degradation. This mechanistic connection builds upon a long history of observations suggesting auxin-like activity for coumarin, from its growth-promoting effects noted in 1959 [6]. to its modulation of root architecture via polar auxin transport [17]. While our work solidifies this link, future research must now focus on elucidating the precise molecular interaction between coumarin and the neddylation machinery to fully understand its allelopathic mechanism.

## 5. Conclusions

In this study, we provide the first evidence that the auxin-resistant mutants of *axr1-3* and *axr2-1* are also resistant to the inhibitory effect of coumarin on primary root growth. Genetic and biochemical analyses revealed that coumarin’s inhibition of root growth depends on the core neddylation enzyme NAE, thereby affecting the degradation of AUX/IAA proteins. These findings establish a close connection between coumarin’s root-inhibiting action and auxin’s regulation of plant growth and development.

## Data Availability

The original contributions presented in this study are included in the article. Further inquiries can be directed to the corresponding authors.

## Supplementary Materials

The following supporting information can be downloaded at https://www.mdpi.com/article/10.3390/biology14121701/s1: Table S1: Primers used in the study; Figure S1: Dose-dependent inhibition of Arabidopsis root growth by coumarin; Figure S2: Semi-quantitative RT-PCR analysis of gene expression.

## Abbreviations

The following abbreviations are used in this manuscript:

*AXR1*	*AUXIN RESISTANT 1*
*AXR2*	*AUXIN RESISTANT 2*
*ECR1*	*E1 C-TERMINAL RELATED*
NAE	NEDD8-activating enzyme
*RUB1*	*RELATED TO UBIQUITIN 1*
IAA7	AUX/IAA 7
NASC	Nottingham Arabidopsis Stock Centre
MS	Murashige and Skoog
DAB	Diaminobenzidine
qPCR	Quantitative real-time PCR
