# Supplementary material for: Coumarin Inhibits Primary Root Growth by Modulating Auxin Signaling via Neddylation
Source: Biology (Basel). 2025 Nov 28;14(12):1701. doi: 10.3390/biology14121701 (PMC12730241; doi:10.3390/biology14121701)
Supplement: Supplementary file 1 [file biology-14-01701-s001.zip › biology-3969169-supplementary.pdf]

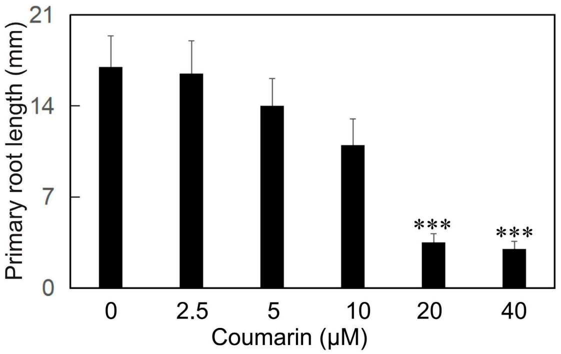

Figure S1. Dose-dependent inhibition of Arabidopsis root growth by coumarin. Primary root length of 7-day-old wild-type Col-0 seedlings grown on 1/2 MS medium supplemented with various concentrations of coumarin. Data are presented as the mean ± SE (n >20). Asterisks indicate statistically significant differences (***, P <0.01).

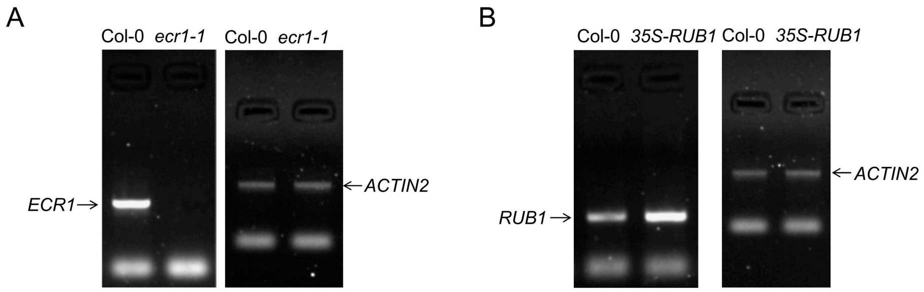

Figure S2. Semi-quantitative RT-PCR analysis of gene expression. (**A**) Semi-quantitative RT-PCR was performed to detect the expression of ECR1 in the *ecr1-1* mutant, with *ACTIN2* as the internal reference gene. (**B**) Semi-quantitative RT-PCR was performed to detect the expression of *RUB1* in the *35S-RUB1* plants, with *ACTIN2* as the internal reference gene.

Table S1. Primers used in the study.

Name	Sequences	Process
ecr1-1-F	ATTGTAAGGGACTGGTAA	Mutant identification
ecr1-1-R	AACTTGTATTGGCTTCTC	Mutant identification
ECR1-RT-F	TGAGGGATGACATAAGGG	Semi-quantitative PCR
ECR1-RT-R	GGTGTAACTCCAGGCAAG	Semi-quantitative PCR
RUB1-RT-F	CAACAAAGGCTAATCTACGC	Semi-quantitative PCR
RUB1-RT-R	CCTAAGGGCAAGAACCAG	Semi-quantitative PCR
ACTIN2-RT-F	GCTCCTCTTAACCCAAAGGC	Semi-quantitative PCR
ACTIN2-RT-R	CACACCATCACCAGAATCCAGC	Semi-quantitative PCR
AXR2-Q-F	GATCCTTCTAAGCCTCCTG	Real-time qPCR
AXR2-Q-R	CTCCTCCACCAAAGTTCC	Real-time qPCR
ACT2-Q-F	GCTGACCGTATGAGCAAAGA	Real-time qPCR
ACT2-Q-R	GATCCACATCTGTTGGAACG	Real-time qPCR
RUB1-OE-F	ATGCAGATCTTCGTCAAAA	vector construction
RUB1-OE-R	TCAGAGAAGACCAAAACCAC	vector construction
AXR2-Flag-F	ATGATCGGCCAACTTATG	vector construction
AXR2-Flag-R	AGATCTGTTCTTGCAGTACTTC	vector construction
